# Peristalsis in the junction region of the *Drosophila *larval midgut is modulated by DH31 expressing enteroendocrine cells

**DOI:** 10.1186/1472-6793-10-14

**Published:** 2010-08-10

**Authors:** Dennis R LaJeunesse, Brooke Johnson, Jason S Presnell, Kathleen Kay Catignas, Grzegorz Zapotoczny

**Affiliations:** 1 Department of Biology, 312 Eberhart Bldg., University of North Carolina Greensboro, Greensboro, North Carolina 27402, USA

## Abstract

**Background:**

The underlying cellular and molecular mechanisms that coordinate the physiological processes in digestion are complex, cryptic, and involve the integration of multiple cellular and organ systems. In all intestines, peristaltic action of the gut moves food through the various stages of digestion from the anterior end towards the posterior, with the rate of flow dependent on signals, both intrinsic and extrinsic to the gut itself.

**Results:**

We have identified an enteroendocrine cell type that regulates gut motility in the *Drosophila melanogaster *larval midgut. These cells are located at the junction of the anterior and the acidic portions of the midgut and are a group of enteroendocrine cells that express the peptide hormone Diuretic Hormone 31 in this region of the gut. Using cell ablation and ectopic activation via expression of the *Chlamydomonas reinhardtii *blue light-activated channelopsin, we demonstrate that these enteroendocrine cells are both necessary and sufficient for the peristalsis in the junction region of the midgut and require the Diuretic Hormone 31 to affect normal peristalsis in this region. Within the same junction region of the midgut, we have also identified morphological features suggesting that this region acts as a valve that regulates the transit of food from the anterior midgut into the acidic portion of the gut.

**Conclusions:**

We have characterized and described a set of enteroendocrine cells called the Midgut Junction DH31 expressing cells that are required for peristaltic movement in the junction region between the anterior portion and acidic region of the larval midgut of *Drosophila melanogaster*. We have shown that the Midgut Junction DH31 expressing cells are necessary and sufficient for motility and that the peptide hormone DH31 is required for peristalsis in the junction region of the midgut. The *Drosophila *model system will allow for a further dissection of the digestion process and provide a better understanding of the mechanisms that regulate digestion in all organisms.

## Background

All higher metazoans have evolved digestive systems that extract sustenance from the environment for growth and survival [1-3]. Although digestive systems are adapted to diverse feeding behaviours, they share an overall similarity in their organization, and all coordinate nervous system and endocrine input to govern the movement and the processing of food within the alimentary canal [4-9]. Central to these concepts is the digestion of food within the gut. In all intestines, peristaltic action of the gut moves food through the various stages of digestion from the anterior end towards the posterior, with the rate of flow dependent on signals, both intrinsic and extrinsic to the gut itself [2,3,10-13]. The exact cellular and molecular mechanisms of these controls, however, often remain enigmatic, and failure of these mechanisms results in inefficient digestion and improper movement of food.

We are interested in understanding the mechanisms that regulate peristalsis of the larval midgut in *Drosophila melanogaster*. The *Drosophila *larval midgut is an endothelial tube composed of two cell types: enterocytes and enteroendocrine cells which arise from stem cell crypts located within the gut and differentiate into either cell type in a Notch signalling pathway-dependent fashion [1,14-17]. Depending on position cues within the gut tube, the enterocytes develop a wide variety of morphologies and functions and represent the majority of the cells within the midgut. While many of the enterocytes are involved in the absorption of nutrients at various stages of digestion, others, such as the acid-producing copper cells within the acid region of the midgut, are highly specialized [1,18-22]. Comprising a smaller portion of the cell population of the gut are the enteroendocrine cells, which have been shown to play several roles including the secretion of a number of peptide hormones and innate immune responses [23-30]. Although many of these neuropeptide hormones are also expressed in the central nervous system where they have been shown to influence behaviour and circadian rhythm [4,9,31], the significance of neuropeptide hormone secretion by gut enteroendocrine cells remains unclear.

The visceral muscles of the *Drosophila *midgut are organized into an inner group of circular muscles that wrap the circumference of the gut and an outer group of longitudinal muscles that traverse the length of the midgut [32-35]. Unlike vertebrate intestines, the visceral musculature of the *Drosophila *midgut is a striated muscle type, most similar in structure to cardiac striated muscle [36] and originate from the same group of dorsal mesodermal cells that give rise to the dorsal vessel, the *Drosophila *heart [37-41].

The foregut, proventriculus, the anterior end of the midgut, and the hindgut are innervated by neurons emanating from the central nervous system [42,43]. Feeding and ingestion of food into the midgut are mediated through interactions of the foregut with the nervous system via the stomatogastric nervous system [3,44]. In contrast to the detailed understanding of the morphology and physiology of the stomatogastric nervous system (SNS) and the foregut, the neural/muscular physiology of the midgut has been less well characterized, despite it being the largest segment of the alimentary canal. Outside of its anterior-most end, the midgut appears to have little connection to the nervous system, and little is understood of the mechanisms that are responsible for maintaining and propagating gut motility and peristalsis through the midgut. In this report, we characterize an enteroendocrine cell type in the *Drosophila *larval midgut that regulates gut motility in the junction region of the larval midgut. These enteroendocrine cells express the peptide hormone DH31, express receptors to acetylcholine, and have stimulatory properties of excitable cell types. Using cell ablation and an ectopic blue-light activation technique, we demonstrate that these Midgut Junction DH31 expressing cells are essential for proper peristalsis within the junction region of the anterior midgut. A mutant analysis of *DH31 *demonstrates that this peristalsis utilizes a novel mechanism involving the secretion of a peptide hormone. We also show that the organization of the midgut junction region is more complex than previously thought. In addition, the Midgut Junction DH31 expressing cells involve the possible coordination of multiple components including a valve, cholinergic AllostatinB/MIP secreting cells, and enigmatic muscle tethers that link this portion of the midgut with the more anterior gastric caeca.

## Results

### Screen of midgut expressing Gal4 enhancer trap lines

**Figure 1:**
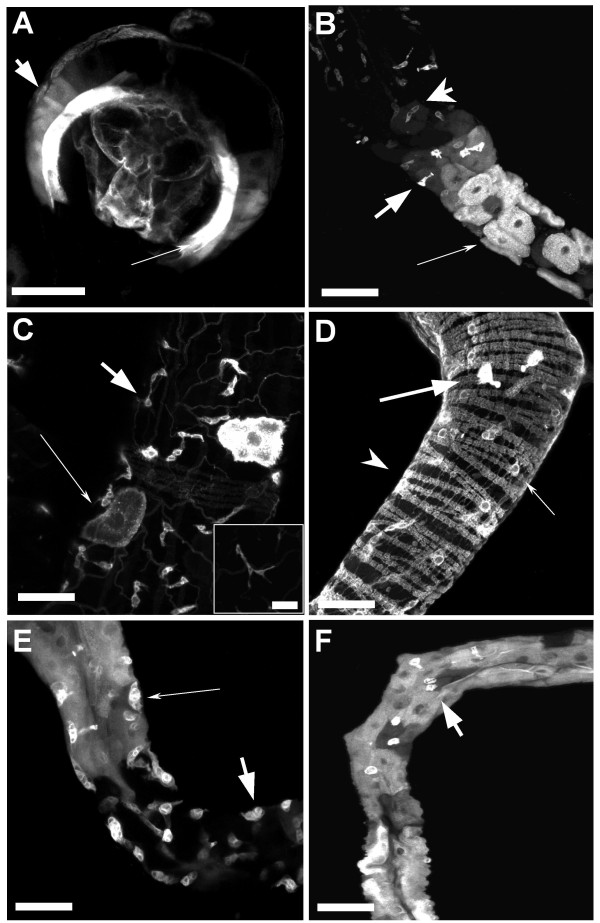
**Gal4 enhancer trap expression patterns in larval midgut**. In all images, the anterior is toward the top and the posterior points towards the bottom. In B-F, the images are of the midgut junction region with the anterior midgut towards the top and acidic region towards the bottom. All size bars are 50 um except for the insert in C which is 10 um. A) The proventriculus is a large bulbous, multilayered portion of the foregut located at the junction of the foregut and midgut. *bric-a-brac *Gal4 expresses in the posterior within the inner layer of the proventriculus (thin arrow) and to a lesser extent within posterior cells of the outer endothelial layer (thick arrow). B) *C135-Gal4 *expresses within the Midgut Junction DH31 expressing cells (thick arrow) and the copper cells of the acidic region of the larval midgut (thin arrow). Expression can also be seen in tracheal precursor cells anterior to the Acidic region (arrow head). C) *DJ717-Gal4 *expresses in tracheal precursor cells (thick arrow and insert) as well as sporadically in the acid producing copper cells (thin arrow). D) *CB30-Gal4 *expresses in Midgut Junction DH31 expressing cells (thick arrow), tracheal precursor cells (thin arrow) and in circular muscles (arrow head). E) *DJ761-Gal4 *expresses in crypt cells (thick arrow) and in anterior midgut endothelial cells (thin arrow). F) *c564-Gal4 *expresses in Midgut Junction DH31 expressing cells (thick arrow) and throughout all cells in the endothelial lining of the larval midgut.

To better understand the different cell types within the larval midgut, we performed a screen of previously identified Gal4 enhancer trap lines and transgenic Gal4 constructs, reported to express in the gut, but which have not been characterized in detail [45-47]. The goal of these experiments was to establish cellular markers to identify different cell types within the midgut, as well as to identify the genes associated with these various cell types, which will provide us with specific molecular tools to manipulate the midgut and better understand how the cells within the midgut interact to regulate peristalsis and the movement of food. Apart from the acid-producing cells and the visceral musculature, the larval midgut consists of several cell types including enteroendocrine cells, enterocytes, and tracheal cells [1,20,24]. Out of the thirty seven enhancer traps screened, nineteen of these Gal4 enhancer traps expressed in various patterns throughout the larval midgut (Table 1; Fig 1).

**Table 1:** Gal4 lines that express in larval midgut

Gal4 Line	Cytological location	Gene/product	Expression Pattern in Midgut
*Cha-Gal4*	3L,91C5	*Choline acetyltransferase*, encodes an choline O-acetyltransferase and is involved in acetyl choline biosynthesis.	MJDH31, VTG
*Ddc-Gal4*	2R, 37C1	*Dopa decarboxylase *encodes an aromatic-L-amino-acid decarboxylase involved in the biosynthesis of serotonin and dopamine.	MJDH31, VTG
*DJ752*	3L, 96F	*Enhancer of Split/HLHm7*	MJDH31
*bab-Gal4*	3L, 61E2-61F1	*bric-a-brac 1*encodes a transcription factor	MJDH31, LM, some CM, some En
*MJ12*	2L, 22B1-22B2	*CG17646*, encodes an ABC type 2 transporter	MJDH31
*DJ761*	3R, 85D16-85D17	*Passila *encodes a nuclear mRNA splicing factor	anterior En
*C805*	2R, 48E6-48E7	*Developmental embryonic B/CG16972, RNA splicing factor*	MJDH31, trach, pattern in Pro
*CB20*	2L, 28B1	*Rapgap1 encodes a *Ras GTPase activator activity;	MJDH31, pattern in Pro
*5053A*	3L, 76C1	*teyrha-meyrah *encodes a protein of unknown function	LM
*Drm-Gal4*	2l, 24C1	*Drumstick *encodes a small C2H2 zinc finger protein	MJDH31, expression pattern in Pro
*DJ691*	3R, 85D22-85D24	*Mura *encodes a zinc finger transcription factor	En and CM
*DJ717*	3^rd ^chromosome		trach, MJDH31, pattern in Pro, Gob, some CM
*CB30*	2R, 56C8-56C9	*Tab2 *encodes a zinc finger transcription factor	CM, MJDH31, En,
*c564*	2^nd ^chromosome	*Insert into repetitive DNA on 2nd*	En
*EDTP/DJ694*	2R, 54B7-54B15	Encodes an egg-derived tyrosine phosphatase	En
*MJ33a*	3^rd ^chromosome		Expression pattern in Pro, MJDH31, FG
*C135*	3^rd ^chromosome		MJDH31, ent, Gob
*T80*	2L, 23A3-23A3	*CG9894, protein of unknown function *	MJDH31, pattern in Pro, weak EN
*T155*	3^rd ^chromosome		pattern in Pro, weak EN

Pro - proventriculus; MJDH31- Midgut Junction DH31 expressing cells; LM - Longitudinal muscles; CM - Circular Muscles; En - endothelial lining/enterocytes; GC - Gastric caeca; Gob -- Goblet cells; VTG - ventricular ganglion, ent - enteroendocrine cells, trach - tracheal cells and precursors.

We found five enhancer traps that express in the visceral musculature. One enhancer trap, *bab-Gal4*, an insertion into the *bric-a-brac *gene, expressed in the proventriculus (Fig 1A), circular and longitudinal muscles (Fig 1C, D), the endothelial lining, and in some enteroendocrine cells. *Bric-a-brac *encodes a transcription factor required for a variety of developmentally important patterning events, including tarsal formation in the leg and terminal filament formation in the ovary [48]. Interestingly, *bab-Gal4 *expressed in the circular muscle, in a pattern flanking the acidic region of the midgut, just upstream and downstream of this region, suggesting that there may be patterning within the circular visceral muscle, perhaps in relation to specific functional regions in the gut. Two other enhancer traps, *DJ717-Gal4 *and *DJ691-Gal4*, are also expressed in a pattern throughout the midgut. One enhancer trap, *CB30*, an insertion into the *Tab2 *transcription factor gene involved in the *Drosophila *innate immune response [23,29], expressed throughout all circular muscles, as well as in the endoderm and a group of enteroendocrine cells (Fig 1D). The *5053A *enhancer trap expressed exclusively in the longitudinal muscles. In this enhancer trap, the *P[GawB] *element has inserted into the *teyrha-meyrha *gene, which encodes a novel protein that has no homologues outside of *Diptera*.

Four enhancer traps expressed in the enterocytes, two exclusively. One of these, *c564-Gal*, is an insert into repetitive DNA on the third chromosome, and the other, *EDTP/DJ694*, is an insert into an egg-derived tyrosine phosphatase [45] (Fig 1F). We also identified enhancer traps that expressed in a pattern within the endothelial lining. *DJ761-Gal4 *expressed only in the endothelium of the anterior midgut, stopping short of the acidic region (Fig 1E). *DJ761-Gal4 *is a *P{GawB} *into the *pasilla *gene which encodes an RNA splicing factor required for normal apical secretion from the salivary glands [49]. Another enhancer trap, *C135-Gal4*, labelled the copper cells of the acidic region, a variety of enteroendocrine cells within the midgut (Fig 1B), and larval fat body. We also identified an enhancer trap, *DJ717Gal4 *that expresses within the nascent tertiary tracheal cells located on the surface of the gut (Fig 1C). These cells are located on the surface of the gut, on top of visceral musculature, and vary greatly in their morphology as they develop into tracheal cells, often appearing round, spindle-shaped, or with multiple projections (inset, Fig 1C).

### Identification of the DH31 Expressing Cells of the Midgut Junction Region

**Figure 2:**
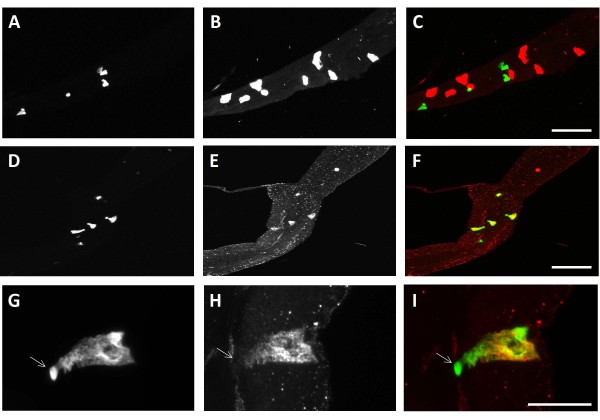
**MIP and DH31 expression in Midgut Junction DH31 expressing cells and junction region**. In A-F, midgut junction regions are oriented with the anterior in the upper right hand corner and the acidic region in the lower left hand side. G-I, the luminal/apical portion of the enteroendocrine cell is on the left and the basal portion of the cell is on the right. The size bars in A-F are 50 um the size bar for G-I is 20 um. A, D, G are DJ752 Gal4/UAS GFP-CD8 reporter gene expression. There are 4 Midgut Junction DH31 expressing cells in (A) and (D). (B) is expression of Allostatin B/MIP in the junction region, where there are eight cells expressing Allostatin B/MIP. C) is the merged image of A and B, note the distinct red and green cells that demonstrate two distinct groups of cells. (E) is the expression of DH31 peptide. (F) is the merged image of (D) and (E). Note that the DH31 expressing cells co-express GFP-CD8 and appear yellow. (G) through (I) are a close-up of the Midgut Junction DH31 expressing cells expressing the DH31 peptide hormone. In (G) the Midgut Junction DH31 expressing cells express the membrane bound GFP-CD8 outlining the cells basal body and luminal apical projection (arrow). (H) All of the DH31 peptide is localized to the basal portion of the cell but not in the apical projection. This is more apparent in (I), with the apical projection shown in green without co-localization of DH31. However, in the basal portion of the cell GFP and DH31 co-localize (yellow).

In twelve enhancer traps/Gal4 reporter transgenes, we observed expression of the GFP reporter gene in a distinct group of enteroendocrine cells located at the junction region of the anterior midgut and the acid-secreting portion of the larval midgut (Fig 2A). This junction region occurs at a conspicuous and characteristic U-shaped bend in the gut, which can be easily identified through the larval cuticle. In four lines, *ChaGal4, DdcGal4, DJ752Gal4*, and *MJ12Gal4*, the CD8GFP reporter expression was observed exclusively within the midgut in these cells (Table 1, Fig 2). The enteroendocrine cells are bottle-shaped and project an apical projection into the lumen of the gut (Fig 2G, I: Additional File 1). We observed an average of 7 ± 2 of these enteroendocrine cells per midgut (n = 37 midguts). Each cell extends through the endothelial epithelium and is associated with the overlying longitudinal muscle.

Two peptide hormones are expressed in two different endocrine cell types within the midgut junction region. One group of midgut junction region enteroendocrine cells exclusively express Allatostatin B/MIP in the midgut [24]; and another group of enteroendocrine cells that are found in this region of the midgut express the peptide hormone Diuretic Hormone 31 (DH31). Other cells in the larvae anterior midgut and hindgut, however, also express the DH31 peptide hormone [24]. The cells were identified in our Gal4 screen expressing DH31 (Fig 2E, F) and do not express the AllostatinB peptide (Fig. 2B, C). These cells will be called Midgut Junction DH31 expressing cells; however not all of the DH31 expressing cells in the junction region express the markers used for the characterization of these cells (Fig 2F). Within the Midgut Junction DH31 expressing cells, the DH31 peptide is located within the cell body, but is excluded from the apical, luminal projection (Fig. 2H, I), suggesting that the secretory portion of these cells is located basally.

### Organization of the Midgut Junction Region

**Figure 3:**
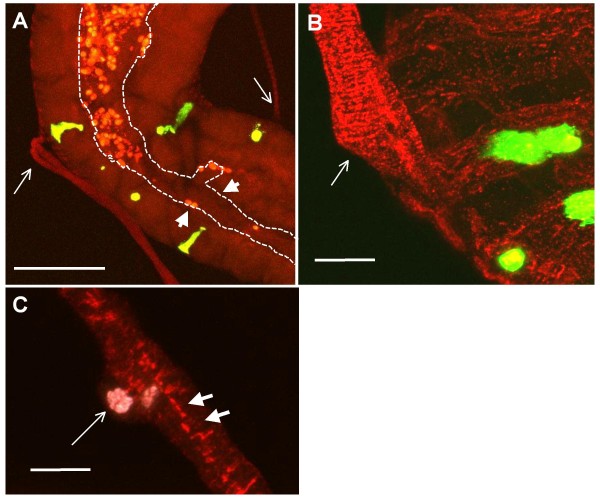
**Organization of the midgut junction region**. A) The anterior midgut is at the top and the acidic region at the bottom. In green, individual Midgut Junction DH31 expressing cells labelled with *ChaGal4 CD8GFP*; in red, actin labelled with Phalloidin Alex 546. The Thin arrows denote the insertion of the muscular tethers within the junction region of the anterior midgut. The arrows heads show the constraint of the lumen within the junction region of the midgut outlined with a dotted line. Also note the yeast auto fluorescence in the lumen as well. Size bar is 50 um. B) Labelling with anti-Discs Large (in red) shows a remnant of the tether (arrow) inserting into the musculature of the anterior midgut junction region. In green (*UAS CD8GFP/DJ752 Gal40*) Midgut Junction DH31 expressing cells are shown. The size bar is 20 um. C) Within the modified longitudinal musculature tether are mono-nucleated (thin arrow) and contain plaques of the Discs Large Protein arranged lengthwise (arrows). The size bar is 20 um.

In addition to the Midgut Junction DH31 expressing cells and the enteroendocrine cells expressing Allostatin B/MIP between the anterior midgut and the acid-expressing cells, we have also found that this junction region contains a pair of muscular tethers that link this region with the gastric caeca (Fig 3A, B, thin arrows; Additional File 2) and a valve (Fig 3A, thick arrows), which manifests as a reduction of the diameter of the gut without a change in the thickness of the endothelial lining. The gastric caeca emerge from the anterior midgut just posterior to the proventriculus as four blind-ended tubes [43,50]. The tips of the ventral gastric caeca are linked to the anterior junction region via these muscular tethers (Additional File 3). The ratio of the diameter of the gut and the lumen at midgut junction region is the smallest within the entire midgut (Fig 3A) and is found at a conspicuous and characteristic bend in the gut. In addition to a bend at this region, the gut also appears to be twisted (LaJeunesse, personal observation). The muscular tethers are extremely labile and typically destroyed during most dissections; however the remnants of which are almost always observed on both the gastric caeca terminus and within the anterior junction region of the midgut (Additional File 4). These muscle tethers express higher levels of Disc Large protein, relative to other visceral muscles, and a preponderance of the Disc Large protein is found in internal plaques along the length of the muscle (Fig 3C). Disc Large protein is a component of the septate junctions in *Drosophila *epithelial cells, a part of the neuromuscular junction bouton, and the internalized plaque structures appear to be distinct from these previously identified subcellular localizations [51,52]. The muscular tethers retain a striated sarcomeric organization of actin and myosin at the attachment to the midgut and at the origin on the gastric caeca. This striated organization, however, becomes less organized within the belly of the tether (data not shown).

### Midgut Junction DH31 expressing cells express AChR and AllostatinB/MIP cells express CHAT

**Figure 4:**
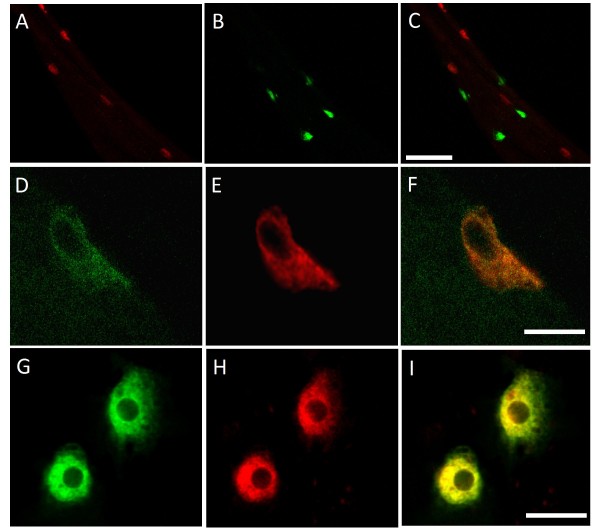
**Expression of CHAT and AChR in the junction region of the larval midgut**. (A-C) Image of a larval midgut at the junction region labeled with *ChaGal UASCD8GFP *and anti-CHAT showing that CHAT is not expressed in the same cells as *ChaGal4*; there is no overlap of the red and green cells in (C). (A) The cells labeled in green are the *ChaGal4 *UASCD8GFP expressing Midgut Junction DH31 expressing cells. (B) The cells in red are cells expressing the CHAT protein. (C) Merged images showing distinct localization of CHAT and ChaGal4 in the junction region of the midgut. Error bar = 100 um. (D-F) Extended View confocal images of the co-localization of CHAT expression with AllostatinB/MIP. (D) Anti-CHAT expression in shown in green.(E) Allostatin B/MIP expression in relabeled with anti-AllostatinB/MIP in red. (F) Merged image shows that the co-localization of CHAT and AllostatinB/MIP. Bar = 10 um. (G-I) Images of living midgut at the midgut junction region. These images are a single plane taken near the basal portion of the cell. (G)The Midgut Junction DH31 expressing cells are labeled with *ChaGal4 UAS-CD8GFP *in green. (H) These same cells are labeled with alpha-bungarotoxin Alexa594 congugate. (I) the merged images show that the labels colocalize. Bar = 10 um.

In addition to DH31, we found that the Midgut Junction DH31 expressing cells express two Gal4 reporters for the genes involved in neurotransmitter biosynthesis, specifically *Choline acetyltransferase *(*Cha*) and *Dopa decarboxylase *(*Ddc*) [53-55]. Since no ventricular ganglion axons project down the anterior midgut into this region, the Midgut Junction DH31 expressing cells are distinct from the nervous system. To determine whether these cells were expressing the enzyme Choline acetyltransferase (CHAT) that is encoded by the *Cha *gene, we examined the localization of CHAT protein in the midgut using a monoclonal antibody [56]. Curiously, although the Midgut Junction DH31 expressing cells express the *ChaGal4 *reporter (Fig 4B), they do not express CHAT protein (Fig 4A, C). This result demonstrates that the *ChaGal4 *transgene does not contain all of the regulatory elements for proper *Cha *expression in the midgut, and its expression in the Midgut Junction DH31 expressing cells appears to be an artefact. However, we observed CHAT expression (Fig 4D) in another group of cells within the midgut junction region, specifically the AllostatinB/MIP expressing cells (Fig 4E, F). Since the MIP/AllostatinB cells appear to be cholinergic, we looked for cells in the junctional region of the midgut that express acetylcholine receptors (AChR) using Alexa594 labelled α-bungarotoxin (BTX-594) which specifically bind nicotinic Acetyl Choline receptors [47,57]. In live Midgut Junction DH31 expressing cells (as shown by ChaGal4 expression of CD8GFP; Fig 4G) we observe binding of the BTX-594 probe (Fig 4H, I); moreover these are the only cells within the midgut which bind this probe. While we haven't been able to find a physical connection or synapse between the Midgut Junction DH31 expressing cells and the AllostatinB/MIP expressing cells, the close proximity of cholinergic cells and cells expressing AChR suggests a functional connection between these two groups of cells.

### Ablation of Midgut Junction DH31 expressing cells/loss of DH31 abolishes normal peristalsis in the junction region of anterior midgut

To determine the role of the Midgut Junction DH31 expressing cells in the midgut, we ablated the Midgut Junction DH31 expressing cells from the larval midgut using a system based on the ectopic expression of either the proapoptotic gene, *UAS reaper*, or the castor bean toxin, *UAS ricin *[58,59]. We directed the expression of these ablation genes using the *Gal80*^*ts *^conditional expression system and two Gal4 Drivers specific to the Midgut Junction DH31 expressing cells in the larval midgut, *ChaGal4 *and *DJ752 *[60]. Larval midguts with ablated Midgut Junction DH31 expressing cells were assessed in four separate assays: (1) a feeding assay to examine the movement of food through the alimentary canal; (2) a morphological assay in which we examined the structure of the midgut; (3) a functional assay in which we directly observed peristalsis within the anterior midgut junction region; and (4) an assay in which the pH of the food in acidic portion of the midgut was assessed using Bromophenol Blue dye.

Although ablation of the Midgut Junction DH31 expressing cells did not change the passage of food through the gut (see Additional File 5) or generate any morphological changes to the anterior midgut junction region (see Additional File 6), we observed significant changes in peristalsis in the anterior midgut junction. We observed a significant decrease in the number of contractions versus the controls (Table 2; compare Additional File 7 with Additional File 8) demonstrating that these cells are central to peristalsis specifically in this region. However, peristalsis while not normal, is not completely lost and gut motility in other regions of the midgut appears to be unaffected. To determine whether the expression of the peptide hormone DH31 play any role in this peristalsis, we examined junction region peristalsis in larvae homozygous for a *Dh31^KG09001^*, a strong hypomorphic mutant allele that contains a P{SUPor-P} insertion into the second intron of the *Dh31 *gene [61,62]. Larvae homozygous for *Dh31^KG09001 ^*have no detectable expression of DH31 peptide as determined by immunohistochemistry (Dennis LaJeunesse personal observation). Again, like the larval midguts from the ablation experiments, larval midgut from larvae homozygous for *Dh31^KG09001 ^*show greatly reduced peristalsis in the junction region of the larval midgut (Table 2; Additional File 9).

**Table 2:** Ablation of the Midgut Junction DH31 expressing cells from anterior midgut junction region alters peristalsis

Genotype	Condition/Temp treatment	n	Average # of contractions per minute ± SD
*w^1118^*	29°C o/n; RT 3 hours	33	10.7 ± 3.1
*UAS ricin/+; DJ752Gal4/Gal80^ts^*	29°C o/n; RT 3 hours	30	2.6 ± 2.8*^A^
*UAS reaper/+; DJ752Gal4/Gal80^ts^*	29°C o/n; RT 3 hours	20	2.0 ± 2.7**^A^
*Dh31^KG09001^/Dh31^KG09001^*	RT	23	1.6 ± 1.6***
*UAS ricin/+; Gal80^ts^/+ (control)*	29°C o/n; RT 3 hours	20	10.2 ± 2.9
*UAS reaper/+; Gal80^ts^/+ (control)*	29°C o/n; RT 3 hours	15	10.5 ± 2.1
*DJ752Gal4/+ (control)*	29°C o/n; RT 3 hours	15	9.6 ± 4.1
*w^1118 ^(wild type)*	RT	43	10.1 ± 3.9
*UAS ricin/+; DJ752Gal4/Gal80^ts^*	RT	20	14.5 ± 5.9
*UAS reaper/+; DJ752Gal4/Gal80^ts^*	RT	20	8.54 ± 4.5

* significance P = 2.5E-16 when compare to w1118 (wild type) 29°C o/n; RT 3 hours control

** significance P = 4.56E-14 when compare to w1118 (wild type) 29°C o/n; RT 3 hours control

***significance P = 1.51E-18 when compare to w1118 (wild type) RT control

*^A ^*- "contractions" in these classes were uncoordinated twitches within the visceral musculature.

o/n - overnight

To further examine the role of the Midgut Junction DH31 expressing cells in peristalsis, we ectopically activated Midgut Junction DH31 expressing cells by expressing the *Chlamydomonas reinhardtii *Channelrhodopsin-2 (ChR2) in the Midgut Junction DH31 expressing cells and examining its effect on peristalsis. The ChR2 protein is a light-activated cation-selective ion channel that, when expressed in an excitable cell type and exposed to blue light (λ~488 nm), will initiate an action potential [63,64]. Using this technique, we showed that the Midgut Junction DH31 expressing cells are not only necessary, but also sufficient for increasing the rate of peristalsis the midgut junction region. We observed a significant increase in the number of contractions in midguts with CHR2-expressing Midgut Junction DH31 expressing cells when exposed to blue light versus white light (+43% for *DJ752Gal4 *and +26% for *ChaGal4*; Table 3).

**Table 3:** Activation of the Midgut Junction DH31 expressing cells is sufficient to induce ectopic contractions in the anterior midgut junction region.

Genotype	n	White light contractions/min	Blue light contractions/min	% change
*w^1118^*	20	12.7 ± 5.5	12.8 ± 2.7	+0.8%

*DJ752 Gal4; UASChR2X2(with retinal)*	20	11.5 ± 4.2	16.4 ± 4.0	+43% *

*Cha Gal; UASChR2X2 (with retinal)*	20	9.7 ± 4.5	12.2 ± 4.0	+26% **

*DJ752 Gal4; UASChR2X2 (no retinal)*	12	11.3 ± 3.6	9.2 ± 3.5	-19%

*Cha Gal4; UASChR2X2 (no retinal)*	14	11.1 ± 4.0	10.3 ± 5.0	-7%

*DJ752 Gal4 (control)*	16	9.1 ± 5.0	9.1 ± 4.5	0%

*Cha Gal4 (control)*	12	9.7 ± 4.3	8.3 ± 3.4	-14%

*UASChR2X2 (control)*	9	11.5 ± 3.6	11.5 ± 3.6	0%

* Significant increase in number of contractions during exposure to Blue Light when compared to the number of contractions during exposure to white light, P = 1.7E-4

**Significant increase in number of contractions during exposure to Blue Light when compared to the number of contractions during exposure to white light, P = 6.4E-4

### Ablation of Midgut Junction DH31 expressing cells and loss of DH31 alters the pH within the acidic portion of the midgut

Since the Midgut Junction DH31 expressing cells are found at valve-like structure poised at the entry of the acidic portion of the gut, we evaluated the pH within this region in midguts from larvae missing the Midgut Junction DH31 expressing cells and in larvae mutant for *Dh31*, with the idea of connecting the defects in peristalsis to some tangible consequence in the processing of food. Typically, larvae-fed food containing 2% Bromophenol Blue will have an anterior midgut with deep blue food and an acidic region marked by bright yellow food with a pH <3.0 [20]. In midguts from Midgut Junction DH31 expressing cells -ablated larvae or mutant for *Dh31 *we observed a significant mixing of acidified and non-acidified food in the acidic compartment of midgut. This result manifests as an increase in the number of larvae with green food instead of yellow food in the acidic region (Table 4). This suggests that Midgut Junction DH31 expressing cells regulate the passage of food into the acidic compartment of the larval midgut.

**Table 4:** Loss of Midgut Junction DH31 expressing cells results in Green food in the acidic region of the larval midgut.

Genotype	Condition/Temp treatment	n	% yellow food in acidic region	% green food in acidic region
*w^1118^*	29°C o/n; RT 3 hrs	16	94%	6%
*UAS ricin/+; DJ752Gal4/Gal80^ts^*	29°C o/n; RT 3 hrs	30	27%	73%
*UAS reaper/+; DJ752Gal4/Gal80^ts^*	29°C o/n; RT 3 hrs	21	38%	62%
*DH31^KG09001^/DH31^KG09001^*	RT	28	36%	64%
*UAS ricin/+; Gal80^ts^/+ (control)*	29°C o/n; RT 3 hrs	11	91%	9%
*UAS reaper/+; Gal80^ts^/+ (control)*	29°C o/n; RT 3 hrs	11	82%	18%
*DJ752Gal4/+ (control)*	29°C o/n; RT 3 hrs	30	97%	3%
*UAS ricin/+; DJ752Gal4/Gal80^ts^*	RT	17	100%	0%
*UAS reaper/+; DJ752Gal4/Gal80^ts^*	RT	14	100%	0%
*DJ752Gal4/+*	RT	12	100%	0%

## Discussion

Although intestinal enteroendocrine cells are a diverse group of cells and play a variety of roles including roles in sensing nutrient levels, immune response to pathogens, and secretion of hormones and neuropeptides [12,24,25,28,30,65-67], little work has been done on show the role of enteroendocrine cells in digestion and gut motility in insects [24,25]. In this manuscript, we have identified a distinct type of enteroendocrine cell within the midgut junction region that we called the Midgut Junction DH31 expressing cells. Ablation of the Midgut Junction DH31 expressing cells from the larval midgut results in abnormal peristalsis in the junction region of the midgut and subsequent inappropriate mixing of acidified and non-acidified food in the acidic region of the midgut. The Midgut Junction DH31 expressing cells express and require the peptide hormone gene *Dh31 *for this peristalsis. While previous work in *Drosophila *has shown that DH31 can stimulate urine secretion from the Malpighian tubules though the activation of a cAMP pathway [4,68], topical application of DH31 has been shown to stimulate contractions in the hindgut and dorsal vessel of the blood sucking insect *Rhodnius prolixus *[69]. Although this data suggests that DH31 stimulates visceral muscle motility whether this peptide hormone directly activates this contraction remains to be tested.

**Figure 5:**
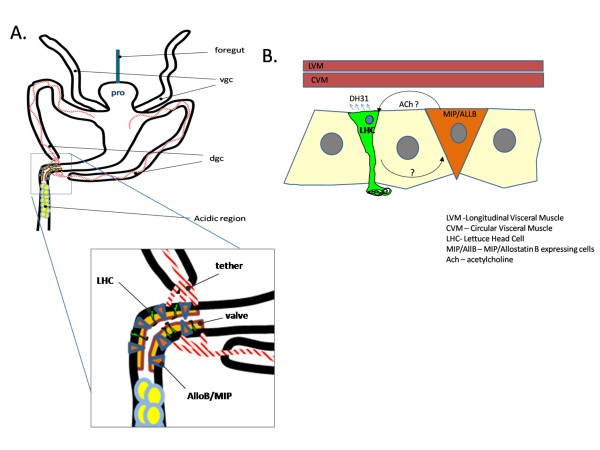
**Organization of the Midgut Junction Region**. A) Schematic of the organization of the midgut junction region. Extensions of the longitudinal muscles (red stripes) of the gastric caeca attached to the midgut junction region which contain two groups of enteroendocrine cells, the Midgut Junction DH31 expressing cells (in green) and cholinergic Allostatin B/MIP expressing cells(mauve outlined/red). The lumen of this region of the gut is thinner when compare to the remainder of the midgut and acts like a valve (in orange). This region of the midgut is a conspicuous bent and twisted before the entry into the acidic region. B) Model of the regulation of peristalsis in the midgut. Midgut Junction DH31 expressing cells secrete DH31 basally to stimulate contractions. This secretion may be stimulated through the atypical ACh paracrine-like signalling from neighbouring Allostatin B/MIP secreting enteroendocrine cells. An unidentified reciprocal signal may emanate from the Midgut Junction DH31 expressing cells to regulate the expression of Allostatin B/MIP or ACh.

The junction region of the midgut contains other features that suggest the Midgut Junction DH31 expressing cells controls a valve that regulates the rate of food input into acidic portion and functions (Fig 5A). The junction region is the narrowest portion of the midgut with a distinctive bend in a twisted gut - a geometry, which perhaps allows for greater control of the flow food through the gut at this point, like a bent/twisted garden hose. In addition to the Midgut Junction DH31 expressing cells, another group of enteroendocrine cells express AllostatinB/MIP. Despite the lack of any apparent physical connection between the Midgut Junction DH31 expressing cells and AllostatinB/MIP enteroendocrine cells, the fact that the AllostatinB/MIP cells are cholinergic and the Midgut Junction DH31 expressing cells express acetylcholine receptors suggests that these two groups of cells may communicate via an atypical, non-neural acetylcholine pathway. There are numerous cell types including skin epithelial cells, immune cells, within the smooth muscle of blood vessels that use acetylcholine signalling to mediate cell shape change and physiological response to various signals [70-72]. This type of signalling is independent of the formation of synapses between cells and has been linked to paracrine, autocrine or even endocrine-like signalling [72,73]. The regulation of peristalsis in the *Drosophila *larval midgut junction region may involve the secretion of ACh from the junction AllostatinB/MIP enteroendocrine cells that modulates the secretion of DH31 from the Midgut Junction DH31 expressing cells (Fig 5B). Conversely, one might imagine that the Midgut Junction DH31 expressing cells may secrete a different signal to regulate secretion of AllostatinB/MIP, which had myoinhibitory activity [74] from neighbouring enteroendocrine cells in the junction (Fig 5B). Questions remain on the potential nature of the luminal signals that regulate this potential cross talk. Both enteroendocrine cells have apical faces that contact the lumen and therefore could respond to specific signals in the lumen to modulate peristalsis. In the human duodenum, enteroendocrine cells called L-Cells regulate the peristaltic movement of food through the gut via the secretion of glucagon-like peptide-1 in response to the presence of glucose [67]. In the case of midgut junction peristalsis, the signal may be a digestive cue in the anterior midgut (i.e. the concentration of some metabolite) that triggers entry of food into the acidic region, or perhaps the signal is a reflux/retrograde signal from the acidic region (i.e. a change in pH) that keeps the valve closed.

Although little is known about the mechanisms that govern midgut motility in invertebrates, there is evidence of coordination between different portions of the gut. Elegant electrophysiological and experimental manipulation performed within the foregut of the blowfly *Calliphora *larvae, which has a similar organization and structure to the *Drosophila *foregut and SNS, has demonstrated that a complex network of interactions among all components of the SNS contribute to characteristic feeding and swallowing behaviours [3,43,44]. Of the SNS, only neurons emanating from the proventricular ganglion innervate the proventriculus and the anterior midgut and the gastric caeca. Interestingly, severing these neuronal connections to the midgut alters contractile activity in the foregut, suggesting that other mechanisms, perhaps of a myogenic nature, are modulating and coordinating the contractions within the foregut with digestive processes further along the alimentary canal[3,43,44]. We have observed coordinated movement within the junction region with feeding behaviour (LaJeunesse unpublished observations), and the muscular linkage between the junction region and the gastric caeca may serve as a physical connection between these two regions of the gut (Fig 5A). These atypical muscular tethers are continuous with the innervated gastric caecal longitudinal muscles and the longitudinal muscles of the gastric caeca are innervated by neurons from the proventricular ganglion[42,43]. This linkage may provide a mechanism for communication between the SNS and the junction region of the midgut. The potential signal may be either mechanical, via a muscle contraction, or electrical, via an action potential. There is a precedent for the latter possibility; in vertebrate hearts, non-contractile muscular Purkinje fibres disperse electrical stimuli from the conduction system to the ventricular cardiac muscles[75,76]. Given the ontological and structural similarities between the vertebrate cardiac system and the *Drosophila *larvae visceral muscle[36,38], this remains an intriguing possibility. Moreover, such a linkage suggests that the entry of food into anterior midgut from the proventriculus and the exit of food into the acidic region are coordinated.

## Conclusions

We have characterized and described a set of enteroendocrine cells called the Midgut Junction DH31 expressing cells that are required for peristaltic movement in the junction region between the anterior portion and acidic region of the larval midgut of *Drosophila melanogaster*. We have shown that the Midgut Junction DH31 expressing cells are necessary and sufficient for motility and that the peptide hormone DH31 is required for peristalsis in the junction region of the midgut. In addition to the Midgut Junction DH31 expressing cells, we have also described several features of this junction region that suggest a new complexity in the regulation of peristalsis in this region including cholinergic Allostatin B/MIP expressing cells, a valve, and a cryptic group of visceral muscle tethers that link the junction region to the more anterior gastric caeca. The movement of food from the anterior midgut into the acidic region of the larval gut represents a single step within digestion, and the ramifications of failure or disruption to these steps remain unclear. Our finding sheds light on the complexity of digestion, its regulation, and suggests a more complex mechanism for larval midgut motility that previously thought. Given its structural similarity to more complex digestive tracts, the *Drosophila *model system will allow for a further dissection of the digestion process and provide a better understanding of the mechanisms that regulate digestion in all organisms.

## Methods

### *Drosophila *Strains

The stocks used in this study are as follows: *w^1118 ^*(wild-type control); P{GawB}DJ752 *(Bloomington stock #8182); *P{Cha-GAL4.7.4}19B (*Bloomington stock #6793); *P{UAS-mCD8::GFP.L}LL5 *(Bloomington stock #5137)*; P{UAS-rpr.C}14 *(Bloomington stock #5824)*; P{tubP-GAL80[ts]}2 (Bloomington stock #7017); P{GawB}bab1[Agal4-5] *Bloomington stock #6802)*; y^1^; P{SUPor-P}Dh31^KG09001 ^(Bloomington stock #16474); *UAS-ricin *(courtesy of K.G. Moffat, University of Warwick); *UAS-ChR2 X2 *(courtesy of Andre Fiala, Department of Genetics and Neurobiology, Theodor-Boveri-Institut, Julius-Maximilians-Universität Würzburg).

### Immunohistochemistry

5-day-old larvae were dissected in 1× PBS containing freshly made 4% paraformaldehyde; larvae were fixed for 3 hours, washed, post0fixed in methanol for 1 hour. Larvae were washed 3× in 1 × PBS, incubated in PBT (1 × PBS/1%BSA/0.1%TritonX-100) for 30 minutes and then incubated overnight in primary antibody diluted in PBT. We used the anti-Disc Large antibody, monoclonal 4F3 (Developmental Studies Hybridoma Bank; 1:1000). The process was then repeated for the secondary antibody using Goat anti-Mouse Cy3 (Jackson Immunological, 115-165-062). For actin visualization, we performed the same procedure without the methanol post-fix and used Alex564 phalloidin (Molecular Probes; 1:2000). For CHAT localization (and any double labelling with anti-CHAT and another antigen) the process was performed as described above except for the following differences: we fixed overnight in 4% paraformaldehyde fixation and we used the ChAT4B1 antibody (Developmental Studies Hybridoma Bank; 1:100[56]). For DH31 and Allostatin B/MIP immunostaining, 5-day-old larvae were dissected in 10× PBS and immediately transferred to a (4% paraformaldehyde + 10× PBS) fix solution. Larval guts were fixed for 2.5 hours, washed six times (30 minutes per wash) in washing solution (0.1% Triton-X, 1% BSA, 10× PBS), then incubated for 1 hour in normal goat serum (10% goat serum in the washing solution) prior to overnight incubation in primary anti-DH31[24](1:500) and anti-AllostatinB/MIP[24] (1:1000). Same washing/goat serum incubation protocol was followed before one hour incubation in secondary antisera. Larval guts were mounted in Dako Fluorescent Mounting Medium. The double labelling procedures followed the protocol used for anti-Chat experiments except the secondary antibodies used where donkey anti-mouse FITC for visualizing CHAT localization (Jackson Immunological,715-095-150) and donkey anti-rabbit CY3 for visualizing AllostatinB/MIP localization (Jackson Immunological, 711-165-152). All micrographs were imaged using an Olympus IX81 inverted FV500 confocal microscope.

### α-bungarotoxin labelling

4-5 day old larvae expressing *ChaGal4 UASCD8GFP *were collected, washed, and dissected in S2 insect cell culture media. Larval midguts were incubated for 30 minutes in a solution of 10 mM solution of α-bungarotoxin Alexa 594 (BTX-594; Molecular Probes, B-13423) in S2 insect cell culture media. To remove excess probe, the larval midguts were washed three times in fresh S2 media for 5 minute each time. Larval midgut were mounted on a clean glass slide and cover slip and viewed using an Olympus FV500 confocal microscope. Images were collected sequentially.

### Ablation/ectopic-activation of Midgut Junction DH31 expressing cells experiments

Flies of the genotype *UAS-ricin *(or *UAS-rpr*) */CyOAct5cGFP; P{tubP-GAL80[ts]}2 *and *UAS-mCD8::GFP.L; DJ752Gal4 *were crossed to each other. In third in start larvae, DJ752 is exclusively expressed in the Midgut Junction DH31 expressing cells of the intestine. Non-Act5cGFP larvae were collected using a Leica MZFLIII Fluorescent Stereomicroscope. Larvae were placed at 30°C for 3 hours to inactivate the temperature sensitive Gal80 inhibitory protein and activate/release the Gal4 expression of CD8GFP and either the cytotoxin gene *ricin *or the proapototic gene *reaper*. Larvae were then removed from the 30°C and reared at room temperature for several hours. Ablation of the Midgut Junction DH31 expressing cells was determined by the early (1 hour) GFP expression and subsequent later loss of expression of the same *GFP *reporter gene. We never saw the appearance of new Midgut Junction DH31 expressing cells as the numbers of Midgut Junction DH31 expressing cells at the one hour stage were always within the normal number found in wild type midguts. During imaging for the movies, the presence or absence of Midgut Junction DH31 expressing cells was determine by observing the junction region under GFP fluorescence illumination - the lack of any GFP expressing cells in this region signified a loss of the Midgut Junction DH31 expressing cells. To image the peristalsis in the junction region, larval midguts were dissected in S2 cell media and mounted on a glass slide with a coverslip with clay feet to prevent compression. A series of 120 images (2 images per second) were captured with UPlanFl20× dry objective using a CoolSnap CCD camera mounted on an Olympus BX51 upright compound microscope. Data was compiled with ImagePro software. Statistical analysis was performed using a two tailed T-Test assuming equal variances in Microsoft Excel. For the gain of function experiments, larvae of the appropriate genotype were cultured overnight on food containing 100 mM all-trans retinal (Sigma, R2500) and imaged the next day as above with one exception: the larvae were imaged under white light for thirty seconds, and then under an oscillating blue light (488 nm; 2 Hz) for another thirty seconds. One set of controls for these experiments was performed as described without the retinal feeding to allow for genotype control.

### Green Food/Yellow Food assay

5-day-old larvae were cultured overnight in food containing 2% Bromophenol Blue, which is blue in basic solution and yellow in solutions with a pH lower than 3.0. Larvae were then dissected and the colour of the acidic portion of their midgut noted.

## Authors' contributions

DRL wrote the manuscript, supervised the research presented, the execution of all experiments and experimental designs described in this paper. BJ performed the Midgut Junction DH31 expressing cells ablation and ectopic activation experiments. JSP and KKC collected confocal images of the Gal4 enhancer traps and the junction region, performed immunostaining, and assisted in the writing of the manuscript. GZ assisted in the generation of movies for the ablation and ectopic activation experiments, as well as the green/yellow food experiments. All of the authors have read and approved the final manuscript.

## Supplementary Material

Additional file 1 **Animation of an individual Midgut Junction DH31 expressing cell**. This animation depicts a single Midgut Junction DH31 expressing cell within the wall of the midgut at the junction region. The apical end is noted by ruffled edge which extends into the lumen of the gut. Click here for file

Additional file 2 **Attachment of the muscular tether of the gastric caeca to the Midgut Junction region**. In this animation of a z-series taken through an intact intestine shows the juxtaposition of a gastric caeca (marked with an arrow) and the midgut junction region, note the presence of GFP expressing Midgut Junction DH31 expressing cells. Higher levels of the DLG protein can be seen in the muscular tether when compared to the remainder of the gut. Click here for file

Additional file 3 **Rotation of the Gastric Caeca with muscular tether attached**. In this animation the muscular tether extends from the tip of the gastric caeca (marked with an arrow). Click here for file

Additional file 4 **Remnant of the muscular tether insertion at the midgut junction region**. In this animation, the connection between the muscle tether and the midgut has been severed leaving only the origin site of the tether in the midgut junction region. The muscle tether branches out and appears to insinuate into the visceral musculature of the midgut junction region which is marked in green by the presence of Midgut Junction DH31 expressing cells. Click here for file

Additional file 5 **Supplementary Table 1, Ablation of Midgut Junction DH31 expressing cells in the anterior midgut does not alter the morphology of the anterior midgut junction region**. A table showing no change in the overall size of the midgut junction region when the Dh31 expressing cells are ablated. Click here for file

Additional file 6 **Supplementary Table 2, Ablation of Midgut Junction DH31 expressing cells does not change speed that food moves through gut**. A table showing the results from a pulse-chase feeding experiment showing no change in the overall movement of food through the gut when the Dh31 expressing cells are ablated from the midgut junction region. Click here for file

Additional file 7 **Midgut Peristalsis at a wild type junction region**. The movie shows the peristalsis within the anterior midgut junction region over the course of 50 seconds (total run time ~1.5 minute). The movie has been looped three times, and the rate increased to 4 frames per second from 2. The region is noted by the remains of the muscular tethers which are projecting from the midgut to the left. Six peristaltic contractions can be observed during this time in this region of the gut. Click here for file

Additional file 8 **Peristalsis in a Midgut Junction region missing the Midgut Junction DH31 expressing cells**. The movie shows the peristalsis within the anterior midgut junction region over the course of 50 seconds (total run time ~1.5 minute). The movie has been looped three times, and the rate increased to 4 frames per second from 2. The anterior midgut junction region is noted by the remains of the muscular tethers which come into view when the midgut rotates during play. In this example, the Midgut Junction DH31 expressing cells were ablated by expression of *UASricin *via the DJ752 Gal4 driver. Although there are no contractions during this period, the midgut does rotate due to gut motility further down the intestine. Click here for file

Additional file 9 **Midgut Peristalsis in the Midgut from larvae homozygous for Dh31^KG09001 ^**. The movie shows the peristalsis within the anterior midgut junction region over the course of 60 seconds (total run time ~1.5 minute). The movie has been looped three times, and the rate increased to 4 frames per second from 2. The anterior midgut junction region is noted by the remains of the muscular tethers which come into view when the midgut rotates during play. As with Additional File 8, there is little motility in this region of the midgut when DH31 is not present. Click here for file
