# Supplementary material for: Peristalsis in the junction region of the Drosophila larval midgut is modulated by DH31 expressing enteroendocrine cells
Source: BMC Physiol. 2010 Aug 10;10:14. doi: 10.1186/1472-6793-10-14 (PMC2933646; doi:10.1186/1472-6793-10-14)
Supplement: Additional file 5 — Supplementary Table 1, Ablation of Midgut Junction DH31 expressing cells in the anterior midgut does not alter the morphology of the anterior midgut junction region. A table showing no change in the overall size of the midgut junction region when the Dh31 expressing cells are ablated. [file 1472-6793-10-14-S5.DOCX]

**Supplementary Table 1:** Ablation of the Midgut Junction DH31 expressing in the anterior midgut does not alter the morphology of the anterior midgut junction region.

| **Genotype** | **Condition** | **n** | **Outer diameter of junction region**  **±SD** | **Inner diameter of lumen**  **±SD** | **Ratio: Outer/Inner diameter**  **±SD** | **Width of intestinal wall**  **±SD** |
| --- | --- | --- | --- | --- | --- | --- |
| *w^1118^* | External Control | 20 | 339.2±52.8 | 123.3±69.3 | 0.38±0.21 | 128.2±51.5 |
| *DJ752Gal4UAS::CD8GFP* | Control | 20 | 341.2±107 | 129.1±60.7 | 0.39±0.17 | 121.9±54.9 |
| *CHAGal4UAS::CD8GFP* | Control | 20 | 268.1±53 | 51.3±64.1 | 0.18±0.21 | 120.1±39.6 |
| *UAS ricin/+* | 30°C o/n; RT 3 hours | 20 | 329.1±69.3 | 204.7±102.5 | 0.59±0.24 | 67.5±26.1 |
| *UAS rpr.c/+* | 30°C o/n; RT 3 hours | 20 | 311.3±71.3 | 169.8±89.9 | 0.52±0.21 | 87.9±34.2 |
| *UAS ricin/+; ChaGal4/ Gal80^ts^* | 30°C o/n; RT 3 hours | 20 | 326.1±116.8 | 152.4±106 | 0.42±0.17 | 95.7±27.4 |
| *UAS rpr.c/+; ChaGal4/ Gal80^ts^* | 30°C o/n; RT 3 hours | 20 | 321.1±85.3 | 148.9±86.9 | 0.45±0.20 | 103.1±39.5 |
| *UAS ricin/+; DJ752Gal4/Gal80^ts^* | 30°C o/n; RT 3 hours | 20 | 372.3±96 | 155.3±64.7 | 0.43±0.16 | 137.6±63.8 |
| *UAS rpr.c/+; DJ752Gal4/Gal80^ts^* | 30°C o/n; RT 3 hours | 20 | 307±83.5 | 124.7±84.8 | 0.38±0.19 | 96.8±30.9 |
