# Supplementary material for: Peristalsis in the junction region of the Drosophila larval midgut is modulated by DH31 expressing enteroendocrine cells
Source: BMC Physiol. 2010 Aug 10;10:14. doi: 10.1186/1472-6793-10-14 (PMC2933646; doi:10.1186/1472-6793-10-14)
Supplement: Additional file 6 — Supplementary Table 2, Ablation of Midgut Junction DH31 expressing cells does not change speed that food moves through gut. A table showing the results from a pulse-chase feeding experiment showing no change in the overall movement of food through the gut when the Dh31 expressing cells are ablated from the midgut junction region. [file 1472-6793-10-14-S6.DOCX]

**Supplementary Table 2:** Ablation of Midgut Junction DH31 expressing cells does not change speed that food moves through gut.

| Genotype | n | % total larvae with blue food in intestine after 2 hour chase of white food | % total larvae with white food in intestine after 2 hour chase of white food |
| --- | --- | --- | --- |
| *w^1118^* | 94 | 61% | 39% |
| *UAS ricin/+; DJ752Gal4/Gal80^ts^* | 81 | 56% | 44% |
| *UAS rpr.c/+; DJ752Gal4/Gal80^ts^* | 83 | 58% | 42% |
| *UAS ricin/+; ChaGal4/ Gal80^ts^* | 97 | 75% | 25%* |
| *UAS rpr.c/+; ChaGal4/ Gal80^ts^* | 96 | 58% | 42% |

Methods: 20-30 4 day old larvae of the appropriate genotype were placed on food containing 2% Bromophenol blue dye overnight at 29ºC. The next morning (8-12 hours later), 20-30 larvae were rested at room temperature for three hours, and then transferred to a white food containing no dye. After two hours of feeding, larvae were scored for the presence/absence of blue food in their guts. * Larvae of this genotype were sick after ablation and showed problems with movement as well as feeding.
